# The Electronic Patient Reported Outcome Tool: Testing Usability and Feasibility of a Mobile App and Portal to Support Care for Patients With Complex Chronic Disease and Disability in Primary Care Settings

**DOI:** 10.2196/mhealth.5331

**Published:** 2016-06-02

**Authors:** Carolyn Steele Gray, Ashlinder Gill, Anum Irfan Khan, Parminder Kaur Hans, Kerry Kuluski, Cheryl Cott

**Affiliations:** ^1^ Institute of Health Policy, Management and Evaluation Dalla Lana School of Public Health University of Toronto Toronto, ON Canada; ^2^ Bridgepoint Collaboratory Lunenfeld-Tanenbaum Research Institute Sinai Health Systems Toronto, ON Canada; ^3^ Department of Physical Therapy University of Toronto Toronto, ON Canada

**Keywords:** eHealth, mHealth, multimorbidity, primary care, usability, feasibility, pilot

## Abstract

**Background:**

People experiencing complex chronic disease and disability (CCDD) face some of the greatest challenges of any patient population. Primary care providers find it difficult to manage multiple discordant conditions and symptoms and often complex social challenges experienced by these patients. The electronic Patient Reported Outcome (ePRO) tool is designed to overcome some of these challenges by supporting goal-oriented primary care delivery. Using the tool, patients and providers collaboratively develop health care goals on a portal linked to a mobile device to help patients and providers track progress between visits.

**Objectives:**

This study tested the usability and feasibility of adopting the ePRO tool into a single interdisciplinary primary health care practice in Toronto, Canada. The Fit between Individuals, Fask, and Technology (FITT) framework was used to guide our assessment and explore whether the ePRO tool is: (1) feasible for adoption in interdisciplinary primary health care practices and (2) usable from both the patient and provider perspective*s.* This usability pilot is part of a broader user-centered design development strategy.

**Methods:**

A 4-week pilot study was conducted in which patients and providers used the ePRO tool to develop health-related goals, which patients then monitored using a mobile device. Patients and providers collaboratively set goals using the system during an initial visit and had at least 1 follow-up visit at the end of the pilot to discuss progress. Focus groups and interviews were conducted with patients and providers to capture usability and feasibility measures. Data from the ePRO system were extracted to provide information regarding tool usage.

**Results:**

Six providers and 11 patients participated in the study; 3 patients dropped out mainly owing to health issues. The remaining 8 patients completed 210 monitoring protocols, equal to over 1300 questions, with patients often answering questions daily. Providers and patients accessed the portal on an average of 10 and 1.5 times, respectively. Users found the system easy to use, some patients reporting that the tool helped in their ability to self-manage, catalyzed a sense of responsibility over their care, and improved patient-centered care delivery. Some providers found that the tool helped focus conversations on goal setting. However, the tool did not fit well with provider workflows, monitoring questions were not adequately tailored to individual patient needs, and daily reporting became tedious and time-consuming for patients.

**Conclusions:**

Although our study suggests relatively low usability and feasibility of the ePRO tool, we are encouraged by the early impact on patient outcomes and generally positive responses from both user groups regarding the potential of the tool to improve care for patients with CCDD. As is consistent with our user-centered design development approach, we have modified the tool based on user feedback, and are now testing the redeveloped tool through an exploratory trial.

## Introduction

### Background

People experiencing complex chronic disease and disability (CCDD) face some of the greatest challenges of any patient population. Patients with CCDD can be characterized as having multiple chronic conditions that impact on their daily lives [1], they are at a higher risk of experiencing poor health outcomes [2,3] and will tend to use health services more than patients with single conditions [4]. These patients are considered to be among the highest cost patient populations in the health care system [2]. Health systems worldwide are examining ways in which care can best be structured to meet the needs of this growing, complex, and high-cost population.

Beyond the challenges that patients face in managing their own illnesses, primary health care providers struggle to manage the multiple conditions with discordant competing symptoms faced by these patients [5] and lack appropriate clinical practice guidelines to guide decision-making [6]. Patient-centered care, which allows for an individualized and holistic approach to patient care, is viewed as crucial to address the highly variable needs of patients with CCDD [7-10]. Patient-centered care can be supported through the adoption of goal-oriented care approaches as a means to help patients prioritize competing issues [11]. However, goals are often not agreed upon between patients with complex care needs and their clinicians [12], and clinicians may consider the process of ascertaining goals to be “too complex and too time consuming” [13].

The electronic Patient Reported Outcome (ePRO) mobile app and portal system were developed to support patients with CCDD and their primary health care providers to collaboratively set and monitor health-related goals. Mobile health (or mHealth) and other eHealth technologies have been previously used to help track health status and monitor symptoms via telemedicine and wearable technologies [14-17] and encourage improved engagement and changes in health behaviors by patients [18,19]. Although there are tools available that help support goal setting and monitoring, most of these are disease-specific (eg, supporting patients with diabetes [20]), and there are few available tools that can address the needs of patients with complex care needs who tend to be high users of the health care system [21].

Development of the ePRO tool was done in collaboration with the technology company, QoC Health Inc. QoC Health Inc. is a Canada-based technology company that is focused on developing patient-centered technology to enable shifting care for patients to the community [22]. The ePRO tool was developed and tested through an iterative user-centered design approach [23]. As a part of this development strategy, conducting usability and feasibility testing is vital to ensure that tools are understandable and can be adopted by target users in typical settings before running larger more costly evaluations [24,25]. This study describes findings from a pilot study to test the feasibility and usability of the ePRO tool from the perspective of patients with CCDD and health care provider users from a primary health care practice in Toronto, Ontario, Canada.

### Feasibility and Usability Framework Guiding Study and Analysis

The aims of this study were to: (1) determine whether the ePRO tool was feasible to be used by patients with CCDD and their primary health care providers as part of the delivery of primary health care services and (2) assess the usability of the ePRO tool from the perspective of both patient and provider. Our emphasis was on exploring these questions in a real-world setting, and as such, we adopted a pilot study approach in which patients and providers used the tool over a 4-week period.

We used the “Fit between Individuals, Task and Technology” (FITT) framework to guide our feasibility and usability assessment [26,27]. The FITT framework suggests that adopting new eHealth systems requires a fit between the user, the technology, and the task or process that is undertaken. Feasibility refers to the ability of users to adopt a technology or intervention in daily routines (often assessed through use of the tool [28]), which is strongly related to the FITT model intention to assess the ability of a technology to be adopted. Usability specifically speaks to how the technology is meeting user needs and tasks. As such, the FITT framework was adopted to assess feasibility overall, with an embedded usability analysis to assess the technology specifically (see Table 1). Tools are typically assessed in terms of efficiency (what resources are required by the user to complete tasks [27]), effectiveness (the ability to complete tasks completely and accurately), learnability (how easily users can learn the system), and user satisfaction with the product [27,29,30]. Using this framework, we sought to answer the following research questions: (1) *Is the ePRO tool feasible to be adopted into inter-disciplinary primary health care practices?* and, (2) *is the ePRO tool usable from both the patient and provider perspective?*

Table 1 summarizes an overview of indicators used to assess feasibility and usability factors. These measures are aligned with similar studies of usability and feasibility [27,29-31]. As is typical in many feasibility and usability pilots, we relied on a relatively small sample size of patients and providers to test the ePRO tool. Although some usability studies have used surveys such as the System Usability Scale [32] or the Post-System Usability Questionnaire [24], given our small sample, we opted to capture most data through qualitative focus groups, interviews, and observational notes. Quantitative data regarding system use were pulled directly from the ePRO system data (ie, information generated from the technology system itself) to capture compliance and adherence information. Qualitative data collection and analysis allowed us to capture the breadth and depth of user experience.

**Table 1:** Feasibility and usability measures.

Conceptual framework		Measure	Data source
Feasibility			
	*User*	Demographics	Patient information form
		Comfort with technology	Patient and provider self-report (in training or in focus groups or interviews)
		Use of technology	Data of ePRO system
	*Task*	Fit into daily routines	Patient focus groups and interviews
		Fit into provider workflows	Provider focus groups
	*Technology*	Usability assessment (in the following category)	
Usability			
	*Efficiency*	Time to complete monitoring (ie, time on task)	System data
		Reported efficiency	Patient focus groups and interviews Provider focus group
	*Effectiveness*	Reported errors	Online *issue tracker* system to communicate errors and problems with the system to QoC Health
	*Learnability*	Reported learnability	Patient focus groups and interviews Provider focus group
	*Satisfaction*	Reported satisfaction	Patient focus groups and interviews Provider focus group

**Figure 1:**
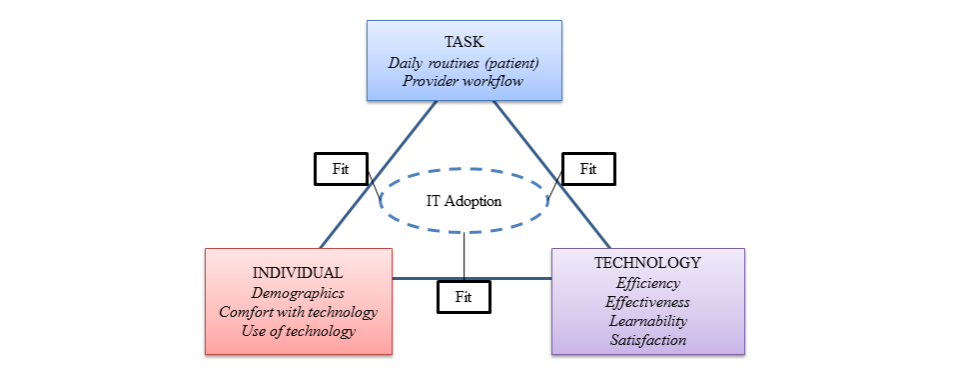
A diagram of the FITT Framework to assess usability and feasibility. Adapted from Sheehan et al [27] p. 364.

Figure 1 presents a diagram of the FITT framework based on Sheehan and colleagues [27] original FITT model which includes the measures used to capture the components of the framework in our study.

In a usability and feasibility assessment, we could have also explored privacy and security issues related to the system [33]. Given our systems are fully compliant with all privacy and security legislation related to transmission of patient data in Canada and the United States, and we had captured patient and provider perspectives on privacy and security in earlier stages of development [34], we did not believe it was necessary to assess privacy and security again at this stage of testing.

## Methods

### Setting

The ePRO tool has been developed within a Toronto-based Family Health Team (FHT); an inter-professional team of providers delivering primary health care services [35]. Patients have a lead primary care provider, either a physician or nurse practitioner; however, patients may receive care from any provider in the team (registered nurses, a social worker, a dietitian, and diabetes nurse educator). Six providers from the team participated in the study as *active ePRO* providers including 1 physician, 1 social worker, 1 registered nurse, 1 dietitian, and 1 diabetes educator. *Active ePRO providers* were involved in goal setting and monitoring of patients at the start of the pilot study and conducted at least 1 follow-up visit with the patient to overview progress using the portal system. Other providers, although not actively participating in the study had the opportunity to view their patient’s data on the portal at any time if they chose.

### Recruitment

*Active ePRO providers* were asked to individually identify 3-5 eligible participants from their practice panels. Eligible patients met all the following inclusion criteria: (1) An FHT patient on the participating provider's practice panel; (2) physical capability to use a tablet or availability of a caregiver who had the physical capability to use a tablet who could enter data on the patients’ behalf; and (3) had complex care needs (2 or more chronic conditions, identified difficulty managing conditions, and social complexity and/or mental health issues) as identified by providers. Potential participants were contacted directly by FHT administrative staff over the phone or when they checked in for their appointment to ask if they were interested in being contacted by a member of the research team at which point they were informed about the study, what would be required for participation, the consent process, and information on privacy of their information. The recruitment process lasted from October 6, 2014 until November 7, 2014; during the process, 12 potential participants were identified. Eleven of the participants could be reached and agreed to participate.

Participants provided informed consent by signing consent forms at the time of training and orientation on the device. Participants also filled out patient information forms at this time to provide us with data on patient demographics. Participants were assured that their participation was voluntary, their personal data would only be accessible by the research team, and he or she could return the device at any time if they became ill or were having difficulties monitoring their goals. All the participants were assigned a unique identifier to ensure their anonymity before data analysis. Online data submitted from the device were on a secure server hosted by QoC Health Inc. The server is compliant with all health information data and security laws applicable in Canada and the United States. The technology partner, who had access to patient data needed to provide technical support, signed a confidentiality agreement before the initiation of the study.

Full ethics approval for this study was obtained from the Joint Bridgepoint Hospital-West Park Healthcare Centre-Toronto Central Community Care Access Centre-Toronto Grace Health Centre Research Ethics Board before the initiation of the study.

### Training

Providers were trained in 2 separate 1-hour group training sessions before the initiation of the pilot study, one session for *active ePRO providers* and another for other providers, mainly physicians from the practice, in the event they wanted to monitor their patients involved in the study. Patients were trained one-on-one with a member of the research team in a 30-minute session at the time when consent was given to participate in the study. Although providing training to users limits our ability to test learnability, patient and provider users required a baseline level of understanding of the tool’s functionality and capabilities to appropriately engage in the tool, allowing us to test other key aspects of feasibility and usability. We anticipate that for this tool to be used as part of regular practice, both providers and patients would require some baseline training; as such, including training in our study offers a closer approximation to real-world use.

### The Intervention: Overview of the ePRO Tool

The ePRO tool includes 2 key features: (1) My Goal Tracker and (2) Hospital CheckOut.

#### Feature #1: My Goal Tracker

The My Goal Tracker feature allows patients and providers to collaboratively identify patient goals and help patients to track outcomes related specifically to those goals. In the development of the ePRO tool, providers indicated that they engaged in goal setting activities with their patients with CCDD as a means to support improved self-management [36]. Activities such as motivational interviewing, counseling, and health coaching are used by providers to help patients manage at home between visits. The tool allows patients and providers to set goals related to 5 different areas identified as most important by patients with CCDD, their caregivers, and their primary care providers in the earlier phases of development. These areas include: (1) maintaining or improving general physical and social well-being (physical health goal); (2) maintaining or improving general mental well-being (mood and memory goal); (3) maintaining or improving mobility (mobility goal); (4) pain management (pain goal); and (5) weight management (diet goal).

Monitoring protocols are linked to each of the 5 goal areas and drawn on the basis of 3 valid and reliable generic outcome measures developed by Patient-Reported Outcomes Measurement Information System (PROMIS). The 3 PROMIS tools that are included are: (1) The General Health Scale; (2) The Pain Interference Scale; and (3) The Health Assessment Questionnaire. The PROMIS tools used in the monitoring protocols are generic rather than disease-specific patient-reported outcome measures to better reflect our patient population of interest. Furthermore, these tools have demonstrated validity and reliability in chronic disease populations [37-39]. Note that the diet goal was not based on a PROMIS tool but rather allowed patients to take pictures of their food and track their weight.

#### Feature #2: Hospital CheckOut

The Hospital CheckOut features allow patients to inform their primary care provider when they have visited and been discharged from a hospital. This feature addresses significant communication challenges identified by patients and providers through a user-needs assessment conducted in the first phase of our tool development [34].The patient simply enters the date of discharge, reason for visit, and name of hospital, and an alert is sent to the provider so that they can reach out to the hospital and retrieve the discharge report.

#### The Portal System

**Figure 2:**
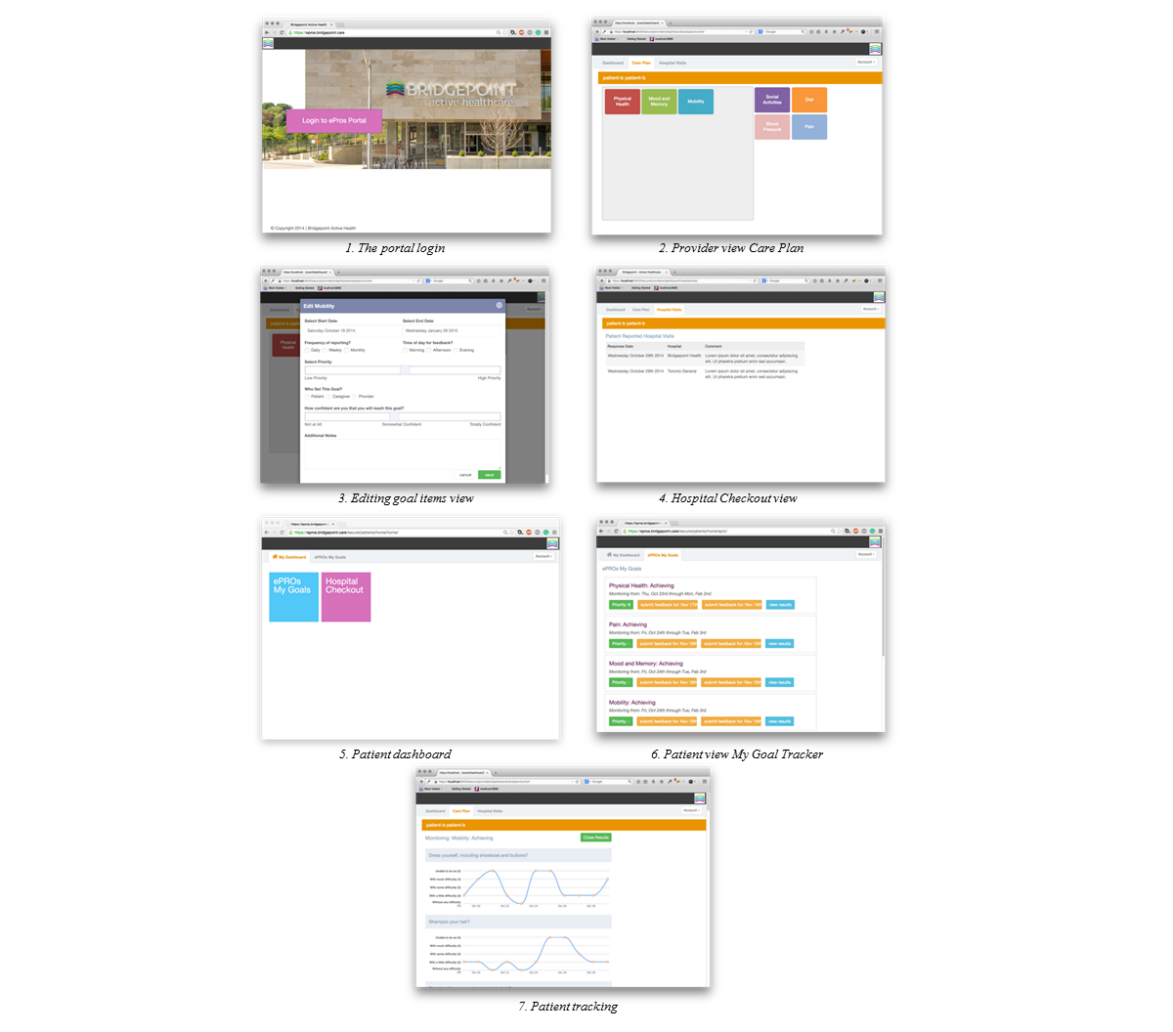
Depiction of the ePRO portal.

The provider portal allows providers to set up *care plans* with patients, which identify on which goals patients will be working toward. Once added to a patients' Care Plan, the goal shows up on the patients “My Goal Tracker,” and the patients then can track their progress toward their goal over time on their mobile device or on the patient portal. The provider is also able to view the Hospital CheckOut alerts on the portal. The patient portal allows patients to view their progress over time. Patients can also choose to enter their monitoring data on the portal rather than on the mobile device if they choose. In general, the portal system was intended to be more heavily used by providers when setting up goal tracking and viewing patient tracking, with patients only needing to access the portal to view their own results; all data tracking activities for patients can be conducted on the mobile app. See Figure 2.

**Figure 3:**
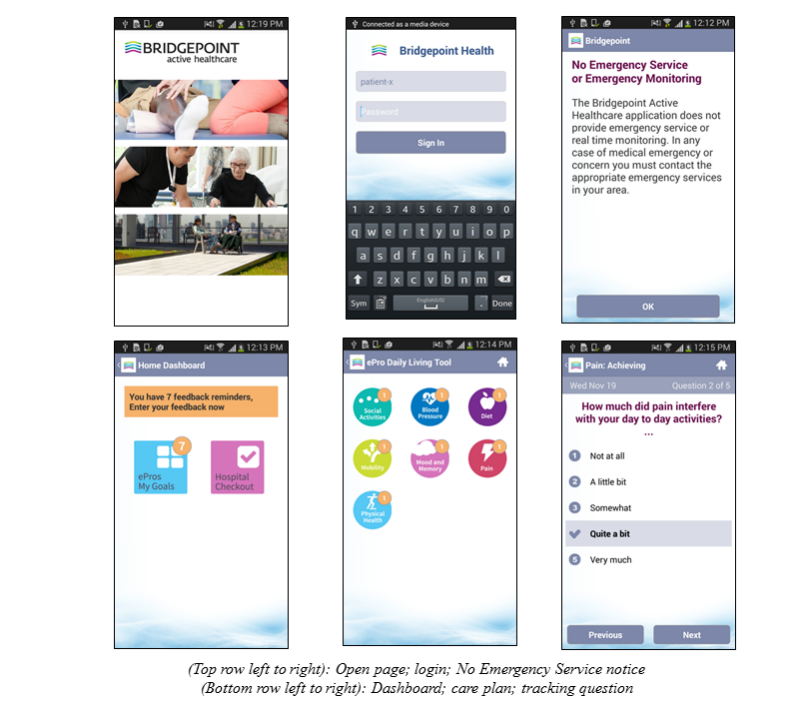
Depiction of the ePRO mobile app.

#### The Mobile App

The mobile app allows patients to track their goals and report hospital visits using the Hospital CheckOut. For the pilot study, patients were provided with locked Samsung Galaxy II android phones with the ePRO app uploaded. As the device was locked, they could only use the ePRO app on the phone. All devices were supported through a fourth generation/long-term evolution connection to not only allow patients to update their data at any time but also allowed the QoC Health Inc. to push bug fixes as required. See Figure 3.

### Pilot

After training, patients worked with providers to collaboratively identify goals and then add them to their care plans. This discussion occurred during a 30-minute appointment, which is typical for allied health professionals working with patients with CCDD to identify goals and improve self-management. Patients then tracked their goals for a 4-week period, after which they returned to their provider to view and discuss their results and tracking. Some patients visited their providers more frequently during the course of the study, which was part of their usual care. For instance, patients visiting the social worker typically visit the social worker once a week, and at each visit, they would discuss goal tracking and modify treatment plans and goals as needed.

At the end of the 4-week pilot study, patients and providers were asked to participate in separate focus groups where they provided feedback on the feasibility and usability of the tool. The patient focus group was held on December 17, 2014 (n=5), and the provider focus group was held on January 22, 2015 (n=6). Three patients who completed the study but were unable to participate in the focus group in December were separately interviewed to gather their feedback on usage of the tool. System data regarding all patient-reported data, types of goals tracked, response counts, time on the system (both patients and provider), and number of times the system was accessed were pulled by QoC Health Inc. and given to the research team in Excel format for analysis, and in addition, they provided an overview report of system use.

### Analysis Method

Basic descriptive statistics were conducted using the system data. Content analysis of data from the issue tracker system was conducted to identify errors reported by users. Focus groups and interviews were analyzed using thematic content analysis [40]. Common themes were identified by 2 different groups of authors, for the patient (AG, AIK, and CSG) and provider (AG, PH, and CSG) focus group data. The authors reviewed the transcripts independently and developed their own preliminary coding scheme. Multiple iterations of coding occurred until consensus was reached on themes among reviewers to develop a final coding scheme [40]. To help organize themes as per the usability and feasibility framework summarized in Table 1, authors (AG and AK) identified coded themes that captured components of the feasibility framework for analysis. Qualitative data management software, NVivo9 [41] was used to manage the data after a final coding scheme was created. Using NVivo 9, researchers code text by highlighting text and selecting the appropriate code from the coding scheme. Coding was conducted by authors (AG, PH, and AIK), and coding reports from NVivo, in which all examples of data attached to a particular code, were generated and reviewed by the lead author along with AG, PH, and AIK to ensure content fit within the definition of the code.

Four patient participants and all providers were willing to provide feedback on the findings (member checking). These individuals were sent packages that included an overview of the analysis and a form to provide feedback. No feedback was returned from patients (1 letter was returned from a patient as the address provided by the participant was incorrect), 2 forms were returned by providers agreeing with findings.

## Results

### Assessing Feasibility: Understanding Users and Tasks

#### Patient User and Tasks

Twelve individuals were identified by providers as potential participants, among them, 11 were successfully contacted by the research team. Participants were both medically and socially complex; they had 2 or more chronic conditions (reported conditions include: diabetes, mental illness, joint conditions, heart conditions, kidney conditions, and chronic pain) and social vulnerabilities (ie, at least 2 of them were in low-income housing; however, socio-economic status was not explicitly captured). Two participants dropped out within the first few days due to health issues, and a third participant dropped out of the study after 2 weeks due to increased anxiety associated with the need to report on her health and using the mobile device. This patient already had high levels of anxiety and depression (including anxiety related to using their personal iPhone) before the initiation of the study. The provider anticipated that this could occur but wanted to give the patient a chance to engage in something new as part of her ongoing treatment. Other studies have found that health monitoring may lead to adverse effects on mental health and well-being [42]. Five of the patients who participated in the full study participated in the focus group, and the 3 others provided feedback through interviews (2 of them over the phone and the remaining 1 patient in person with CSG). See Table 2 for an overview of patient users and their use of the ePRO tool during the pilot.

**Table 2:** Patient demographics, goal monitoring activities, and system usage.

Patient participants		N=11
	Average age	58 years
	Min	35 years
	Max	72 years
	Male	n=5
	Female	n=6
	Other (ie, transgender)	n=0
		
Country of origin		
	Canada	n=5
	United Kingdom	n=2
	United States	n=1
	Jamaica	n=1
	Not reported	n=2
Reported comfort with technology		
	Previous experience	n=6
	Little experience	n=2
	Not reported	n=3
Attrition		n=3
Goals tracked		
	Physical health	n=6
	Mood and memory	n=3
	Pain	n=2
	Diet	n=2
	Mobility	n=1
	Patients tracking 1 goal	n=4
	Patients tracking 2 goals	n=4
	Patients tracking 3goals	n=1
	Patients tracking 4 goals	n=1
	One dropout patient did not set a goal	
System usage		
	Unique survey completions	210
	Questions responded	1311
	Portal access (only accessed by 4) patients)	1.74 average Min 0 Max 3

**Table 3:** Time to complete monitoring tasks for patients.

Goals	No. of questions per survey/protocol	No. of protocol completions	Time to complete protocol (minutes)
Physical health	6 required, 3 optional	75	Average: 3 Min:1; Max:12 1 outlier: 1428^a^
Mood and memory	5 required, 3 optional	68	Average: 5.3 Min:1; Max:57 2 outliers: 1425 and 1180^a^
Pain	5 required, 3 optional	52	Average: 5.6 Min: 1; Max: 31 1 outlier: 1427^a^
Mobility	23 required, 2 optional	3	Average: 6.3 Min: 2; Max: 16
Diet	2 required, 1 optional	9	Average: 1.7 Min:0; Max:3 1 outlier: 683^a^

^a^If patients left surveys in the middle of completing them, the system recorded the full amount of time it took to complete the survey resulting in outliers as high as 1428 minutes.

Patients reported that they manage their multiple chronic illnesses, acute issues that arise, and personal issues as part of their daily lives. Patients also reported using other apps to help manage their health with activity apps and tools such as FitBit, JawBone, and blood sugar monitoring apps.

#### Provider User and Tasks

*Active ePRO providers*, differed in terms of profession and the role they played in the primary care practice; however, all engaged in goal-setting activities with patients before the initiation of the pilot study. Through training and piloting, it was clear that most of the provider participants had a strong competency with technology with only 1 participant identifying more limited capability. In general, providers work in a busy practice where they typically get 30 minutes with their patients, whereas the physician gets closer to 10 minutes. Providers accessed the portal much more often than the patients, on average 10.4 times over 4 weeks (min: 4, max: 15), which is understandable as patients later reported forgetting that there was a portal or experiencing errors with the portal.

Providers reported that they were primarily only able to view patient data just before a patient’s follow-up visit to see a snapshot of the patient’s progress between appointments as that fit more readily into their existing workflow. Some providers reported seeing patients weekly (as was the case for the social worker), and others saw patients more on an as-needed basis. Providers noted that they already engaged in goal-setting activities with their patients often using the SMART goal framework (used initially in rehabilitative settings, see [43]). The practice also provides chronic disease management programs (ie, smoking cessation program). All providers are required to chart all patient encounters in their electronic medical record (EMR).

### Assessing Usability: Technology Assessment Efficiency, Effectiveness, and Satisfaction With the ePRO Tool

#### Patient Feedback

##### Efficiency

Although each protocol did not take long to complete (on average 2-6 minutes, see Table 3), patient participants found that data entry became a tedious task, and at least 2 participants noted that they began completing questions for multiple days at one time, potentially compromising the validity of the self-report data. The ePRO system data revealed that in fact 5 patients completed questions for multiple days at one time, which accounted for between 8% and 46% of the participants’ answers. Participants also found that they forgot to do their monitoring when managing acute health issues or other life stresses. For example, one participant was managing chronic pain issues and lapses in her memory and experienced difficulty incorporating the device into her daily life.

And I don’t think that the pain was the total cause of memory lapse but it had a great deal to do with it.I mean I was going through a great deal of stress. So when I started to add them all together, you know, the stress, the workload, the impending developments that were happening in my life in completely different areas, you know, it was just like I'm standing here trying to juggle 8 balls without having any of them touch the floor it has a tendency to make you forget about whatever else you're doing

P009

atients made a number of suggestions for creating a more responsive tool that would complement their daily routine through reminders (for patients to input their data) and feedback acknowledging their completion of questions, flagging results (as appropriate), and connecting with the other health monitoring apps (ie, FitBit, JawBone, blood sugar monitoring). The most common suggestion was to enable connections between their health goals from the ePRO tool with other apps they were using to improve efficiency of their self-monitoring efforts.

Like linking with FitBit, for example... Yes, I think that would be very useful. Because your…well, it depends on how you use FitBit of course but, you know, you are tracking a lot of indicators around activity, weight, etc…. if you ’re diabetic, is it to link your blood sugar to your performance? You know, all those kinds of things. I think it would have to have a specific reason for using it.”

P002

##### Effectiveness

Some patients in the study identified that they felt using the tool had an early impact on their ability to self-manage and track their health goals. The tool helped to catalyze a sense of responsibility over their care and helped to improve patient-centered care delivery.

I knew why I felt better one week and why I did not feel better the next week.

P011

[my provider and I] were able to see …that [my goal] was not moving really, and to try to change it better…

P005

Patients were able to reflect on their progress of achieving their goals with providers and valued this interaction.

When we were talking about my goal, we were going through the charts, her giving her opinion. She was actually changing my opinion of how I should have answered that question. Well, that should be more interactive. I’d like to get her to do that sooner.

P012

However, patients experienced technical errors and difficulty in reviewing previously entered open-text data. In addition, questions often did not appear at the correct times or days, and patients were unable to locate different features on the tool. One patient and provider were so frustrated by the errors that they nearly pulled out of the study. This particular patient called both the provider and research team frequently to troubleshoot connectivity issues and to discuss aspects of the tool they did not like.

##### Learnability

The learning process was also an important challenge as one-time training was not considered to be sufficient. Participants suggested that adding instructional videos to highlight the key steps for using the device might be beneficial for visual learners and easier to follow than written instructions.

##### Satisfaction

Once technical errors experienced in the first 2 weeks of the study were resolved, patients appreciated having a device to help them monitor their health goals and health behaviors.

I found it very easy to use. I found that once I determined when I was going to do it daily, that I did it. I always got it done

P002

However, some patients reported feeling isolated with the mobile device, and felt that the tool could become a replacement for inperson consultation.

I think the only concern that I had big time was that it might be used to replace people. And that would really sadden me. I think especially in this time and, you know, people do really need people-to-people care and contact. And especially when they’re sick

P003

To mitigate feelings of detachment from the health care provider, participants suggested that having questions and goals that were more specific to the participant’s conditions, as well as personalized feedback from the provider or peers from the clinic would be helpful. Tool-enabled feedback, particularly from peers, was also viewed as a way to offer encouragement on progress toward goal attainment by way of a shared experience.

#### Provider Feedback

##### Efficiency

The research team was unable to track the time it took for patients and providers to set up the goals and the monitoring regime. Providers would schedule a 30-minute session to complete this task; however, they reported that the actual use of the tool would not take the full 30 minutes, but often the conversation about goal setting with the patient would require the entire visit.

After the initial visit, provider participants reported experiencing difficulty incorporating patient data into their workflow in terms of: (1) increased charting time required to input data into the provider’s EMR and (2) being able to view data in manageable chunks.

It would be very helpful if I can see that reporting for a week. Because what I did was I chose people that I see weekly anyway. But realistically in terms of workload even one patient, I bet I would not look at it, you know, every day.

Provider 06

Providers also identified a desire to have the content of the tool better aligned with current programs and practices. As one provider noted:

You know, in terms of using that in the template, that kind of way to set up and frame a goal with them... Because we're working with SMART goals, they need to be that specific...

Provider 04

Finally, some providers felt that they may be liable for monitoring patients during out-of-office hours, which would require additional time and resources. One provider discussed a specific experience during the pilot in which one of her two patients enrolled in the study started “sinking fast,” which she was able to catch by reviewing monitoring data on the portal. However, the provider identified this may not be a scalable solution in future:

Oh my god, this guy is sinking fast, I better make the phone call. Which I did. But I can’t do that for all my patients all the time.

Provider 05

Although it was made clear to providers and patients in training and on the app (see the “No Emergency Monitoring” message in Figure 3) that continuous monitoring was not available, some providers still reported experiencing conflict between ensuring that the tool fit in their existing workflow and their desire to provide high-quality care to their patients. Over the course of the pilot providers, most of them reviewed data before the patient’s appointment rather than monitoring in-between visits and found greater potential for the data to be useful as a synopsis of the patient’s progress.

##### Effectiveness

Some providers noted that having the tool in front of them helped to focus the conversation on patient goals.

To be able to talk about this with her, it was like, oh, thank God I can help set a goal.”

Provider 06

However, providers had difficulties goal setting for some patients, as the goals on the tool were not specific to the health monitoring options on the tool. Providers expressed concerns that the tool’s content was not comprehensive, as setting a specific goal and self-management were two different elements for chronic disease management:

Well, because we’re diabetes educators, R2 and I, we set goals all the time. But the goals that we’re setting were not reflecting in the options that were available. So there was no blood sugar. You know, there's no goal for blood sugar. There was no goal for pain management. There was no… Like there were n’t specific… They weren’t specific enough. And because we’re working with smart goals, they need to be that specific.

Provider 05

To improve effectiveness (and efficiency), providers wanted the tool to fit better with their existing workflows and programs, for example, through better alignment with creation of SMART goals for patients or allowing for monitoring protocols that aligned with goals of existing chronic disease management programs.

Providers experienced technical issues with the device and Web portal, including data not appearing on the portal after being entered on the mobile device, goals not showing up on patients' devices after being set up on the portal, and some challenges with login.

##### Learnability

Providers were unsure of where in the tool to enter free text and how to locate specific goals within the tool. In the focus group, providers noted forgetting about certain elements of the tool, suggesting the need for a manual or additional training sessions.

##### Satisfaction

Although providers also recognized the value of the tool in assisting the patient to self-monitor their chronic conditions, self-monitoring and goal attainment were two aspects in which the tool was felt to be lacking. For example, one provider discussed his or her goal of helping improve the patient’s mood and using the tool to monitor the patient’s eating habits and how the tool offered limited utility in supporting that goal.

The mood one might be a good pre and… Like I do n’t know, it depends on what the goal is. But like maybe a pre and post, doing the study to get like a sense of where they are before and where they are after. But like again with like eating the piece of fruit, her answering those questions every day, I do n’t know, maybe she gave you feedback saying that was really helpful, I do n’t know. I didn't think it was all that helpful. At least it wasn't helpful from my perspective.

Provider 04

With regard to goal attainment in particular, providers were concerned that questions were not specific enough to match goals.

### FITT Assessment of the ePRO Tool

The technology assessment (efficiency, effectiveness, learnability, and satisfaction with the tool) was compared with identified user needs and tasks. This analysis is summarized in Table 4 to provide an assessment of the feasibility and usability of the ePRO tool.

**Table 4:** Feasibility and usability assessment overview.

	Users	Task	Technology
Patients	Multiple chronic conditions with moderate comfort with technology. Interests in monitoring goals related to physical health, mood and memory, pain, diet, and mobility Primarily used the mobile device	Daily routines: Have multiple health and personal concerns to manage Some already using other self-management support tools and apps	The ePRO tool met user needs to monitor and track goals they wished to work on; however, it did not fit well with daily tasks given questions were repetitive and not appropriately tailored to goal activities, and the tool was unable to connect with other monitoring activities in which patients were already engaged.
Providers	Multi-disciplinary providers from primary health care practice, moderate to high level of comfort with technology. Busy practice with limited time to monitor patients between visits. General interest in helping patients better manage	Workflows: Only able to review data before visit to get a snapshot view of the patient, limited time to monitor patients in-between visits Use SMART goals Need to chart in EMR systems	The ePRO tool was helpful in getting patients to discuss goals as a strategy to improve management; however, it did not fit well with provider workflows in terms of supporting SMART goal development and integration with the EMR.

## Discussion

Despite the many challenges, patients and providers demonstrated near-daily use of the device over a short period of time. When we explore some of the more detailed feedback from the focus groups, ease of use of the tool, the noted impact on ability of patients to self-manage and patient-centered care delivery, and the potential for the tool to improve on their sense of responsibility over their care, may be the reason why there was daily usage among patients and weekly usage among providers despite challenges. Providers were equally positive about the potential of the tool to improve efficiency and patient-centeredness at the point of care, particularly if suggested changes were to be implemented.

However, based on our assessment criteria, overall feasibility and usability of the tool are determined to be low from both the patients’ and providers’ perspectives. Concerns regarding the impact on patient-provider relationships, the repetitive nature of questions leading to individuals filling out multiple questions at one time, and an inability of the system to connect with other monitoring activities they were already doing (eg, physical activity monitoring using other apps and devices) were among the more notable issues identified. Furthermore, the system was plagued by connectivity errors, which caused ongoing concerns and frustrations for both patient and provider users. Errors in usability tests are not uncommon and have been noted to impact on usability [17]. We should also consider that daily data entry may be too frequent for most users, and as such, looking for ways to collect data unobtrusively (eg, through connecting to a FitBit or JawBone device) may not only meet identified user needs but could also serve to reduce respondent fatigue.

Providers had a difficult time integrating the monitoring into their daily workflows. Although these providers were already doing goal setting with patients, concerns with a lack of integration with their EMR system and content that did not follow their usual model of care made usability and feasibility of the ePRO tool challenging. A notable concern is how the use of standardized monitoring protocols did not allow for effective monitoring of patient goals, which speaks to a relatively poor task-technology fit. Although the PROMIS tools are valid and reliable measures, these types of generic outcome measures are less helpful for day-to-day management of health-related goals for patients with CCDD. The adoption of valid and reliable patient-reported outcome measures thus may not be as useful in ongoing management of patients with CCDD and patient-centered care delivery [44] but rather may be more useful to assess whether goal attainment is having an effect on outcomes over the medium to long term (6-12 months).

Workflow integration is an important consideration as the introduction of technologies not only augments work processes but in fact reorganizes them [45]. Coupling ehealth tools successfully to health care provider workflow is challenging. Heterogeneity of care practices paired with interdisciplinary team roles and responsibilities, as with our FHT providers, makes it increasingly difficult to accurately and consistently predict provider care needs, especially when caring for patients with complex care needs [46-48]. However, the appetite for mobile health solutions that match provider workflow is in demand [49], with the likelihood of meaningful adoption increasing when end users are involved in the design process [50].

Unfortunately, the tool did not support interdisciplinary practice as we had hoped. We had anticipated that data could be used by the entire FHT (not just the active providers) to help in management of patients; however, the tool did not get used in this way during the pilot study, rather, providers used data in their own management of patients as part of their solo practice. Four weeks may have been a too short period, and potentially, longer term use may provide more opportunities for the data to be used by the interdisciplinary team. The impact on interdisciplinary practice will be examined in our exploratory trial (4-month trial) and pragmatic trial (12-month trial) of the ePRO tool.

The low usability of the tool was a somewhat surprising finding given the extensive user-centered design approach used in the earlier phases of this study. Providers from the FHT as well as patients and caregivers provided detailed and ongoing feedback on the development of the tool (outlined in [36]) before running the usability pilot; however, many of the usability and feasibility challenges experienced in the pilot presented here, particularly those with regard to content, were not identified in earlier stages of development. Typical user-centered design approaches may face challenges when addressing particularly complex patient populations, such as children and those with cognitive impairment, and user feedback may not be useful in all phases of the design process due to the lack of necessary design skills in users [51]. More than likely, however, was that the challenges experienced were not foreseen, and as such reinforced the importance of conducting small, real-world setting pilots such as those presented here as part of a user-centered development approach.

### Limitations

Quantitative data on usage pulled from the ePRO system data did not provide the level of detail we would have liked with regard to tool efficiency. We had made a deliberate decision to do a real-world pilot test of the tool rather than test usability in a laboratory setting as has been used in other usability tests [52]. Although limiting, we dictate that real-world usability testing was more valuable when developing tools for a diverse user group such as patients with CCDD and multidisciplinary providers. Future testing will seek to refine the data that can be collected from the ePRO system to be able to extract time data more effectively. Another limitation of our study was the attrition rate of the patient participants. One of the challenges working with patients with CCDD means that health issues may impede ongoing participation in research. Future piloting and evaluation work will seek to oversample to a greater extent to offset likely dropouts.

Although the numbers of test users are low in our study, Nielsen and Landauer’s [53] model of detecting usability problems suggests that most usability problems (75%) can be detected by 10 users, and 50% of them can be detected by as few as 5. Furthermore, they offer a cost-benefit analysis of including additional users in testing and suggests that an optimal number of test subjects in medium-large projects is 6.7 when using test users to conduct a usability assessment. As such, although our numbers are low, they are aligned with other similar usability studies [30,31,54-57] and also allow us to capture most usability problems we are likely to see.

Finally, we were only able to conduct focus groups and interviews after the pilot study rather than during the study. In a similar pilot, Verwey et al [17] interviewed each user directly after all consultations to capture usability information, which may allow for capturing more useful data.

### Conclusions and Future Works

Although our study suggests relatively low usability and feasibility of the ePRO tool, we are encouraged by the early impact on patient self-management and patient-centered care delivery as well as the general positive response from patients and providers regarding the importance and potential of a tool, which supports goal-oriented care delivery for patients with CCDD in primary health care settings. Findings from the pilot will be used to modify the ePRO tool to improve its feasibility and usability. Key considerations moving forward include: modifying goal monitoring protocols to allow for tailoring to specific goals, ensuring that we target patients with CCDD who could benefit most from goal-oriented care and prescreening for those who may be likely to experience anxiety related to participation, integrating the system into the EMR or with provider workflows, enabling integration with external apps used by patients (eg, JawBone or FitBit), and providing ongoing training opportunities potentially embedded within the tool (ie, through videos and walk-throughs).

## Abbreviations

CCDD: complex chronic disease and disability
EMR: electronic medical record
electronic medical record: electronic Patient Report Outcomes
FITT: fit between individuals, tasks, and Technology
IT: Information Technology
PROMIS: Patient-Reported Outcomes Measurement Information System
SMART goals: Specific, Measurable, Achievable, Realistic, Timed goals
